# Dynamic sensor adaptation based on efferent feedback for adaptive bio-inspired sound source localization

**DOI:** 10.3389/fnins.2026.1736957

**Published:** 2026-01-28

**Authors:** Steve Durstewitz, Daniel Schmid, Timo Oess, Hesan Ghazanfari, Heiko Neumann, Marc O. Ernst, Claudia Lenk

**Affiliations:** 1 Group of Biomedical Sensor Systems and Microsystems, Universität Ulm, Ulm, Germany; 2 Institute of Neural Information Processing, Universität Ulm, Ulm, Germany; 3 Applied Cognitive Psychology, Universität Ulm, Ulm, Germany

**Keywords:** dynamic sensor adaptation, efferent feedback, interaural level differences, lateral superior olivary complex, neural network, neuromorphic computing, recurrent processing, sound source localization

## Abstract

Auditory perception and localization are fundamental tasks for many species, allowing them to detect, identify, and spatially localize sound sources in their environment. While biological systems have evolved sophisticated neural mechanisms for auditory adaptation, artificial auditory systems still struggle to match their performance, particularly in dynamic and noisy environments. Our research focuses on whether sensor adaptation, driven by efferent feedback from the processing stage to the sensory stage, can improve localization performance. Inspired by human sound source localization based on interaural level differences (ILD) and efferent feedback, the proposed neuromorphic system architecture is composed of two bio-inspired acoustic sensors connected to a neural processing stage, represented by two neurons of the medial nucleus of the trapezoid body (MNTB) and two neurons of the lateral superior olive (LSO). The LSO neuron response was analyzed in the following ways: (i) using measured sensor responses at different ILD without efferent feedback and with a fixed local feedback for each sensor measurement; (ii) simulated with synthetically generated sounds with varying ILDs for four different feedback configurations from the LSO neuron to the acoustic sensors. Results from (i) showed how the feedback tuning can be used to overcome mismatches due to fabrication tolerances between different MEMS sensors, and (ii) showed the influence of different feedback configurations and simulation parameters on the LSO neuron response with respect to different ILDs.

## 1 Introduction

Localizing, segregating, and identifying different sound sources in natural sound scenes are extremely difficult tasks, yet animals perform them effortlessly in their everyday lives across a wide range of environments and a varying number of sound sources (Bregman, 1990). The human auditory system, in particular, exhibits remarkable capabilities, enabling individuals to adapt to and focus on specific sound sources even in noisy conditions – a phenomenon known as the “cocktail party effect” (McDermott, 2009; Cherry, 1953). This impressive ability relies on two mechanisms: first, combining the outcomes from different processing tasks, such as sound recognition and identification (e.g., speech and traffic noise), which improves speech-in-noise perception, and second, adaptation of signal sensing and processing. Machine hearing, in contrast, struggles strongly with sound-in-noise perception (Mawalim et al., 2024; Kumar et al., 2024). Thus, we present a bio-inspired approach and its neuromorphic implementation that combine adaptation and binaural sound-source localization to improve machine hearing for sound-in-noise perception.

**Figure 1:**
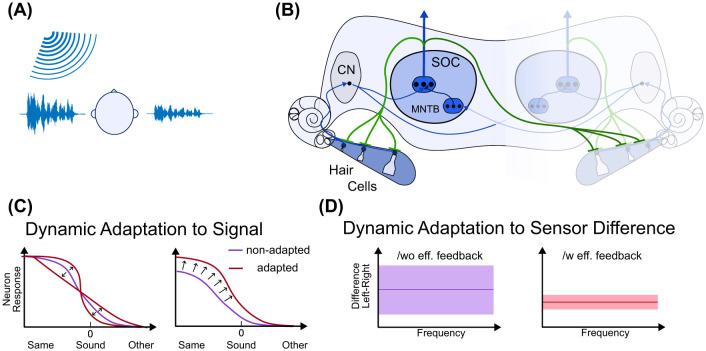
Efferent feedback and sound source localization. **(A)** Interaural level differences (ILDs), shown by the signals, due to the head shadow. **(B)** Schematic representation of stages of human ILD analysis and efferent feedback to the cochlea. Hair cells transduce sound waves into electrical activation on the auditory nerve. These signals pass through the cochlear nucleus (CN) and are then forwarded either to the superior olive complex (SOC) or the medial nucleus of the trapezoid body (MNTB). The SOC output is forwarded to additional brainstem areas, such as the inferior colliculus (not shown). Efferent connections (green lines) stem from the SOC and can be ipsi- or contralateral connections to the hair cells and auditory nerve fibers in the cochlea. **(C)** Dynamic adaptation (purple and dark red curves) due to either local feedback and modulation or efferent feedback can yield a change in sensitivity (slope of response) or ILD range with the highest response (or sensitivity). **(D)** Dynamic adaptation can decrease the influence of sensor differences and balance the left and right input to the SOC or higher brain stems to improve perceptual sensitivity to ILD. In this case, the adaptation is mainly driven by efferent feedback.

Binaural hearing is a key factor in improving speech recognition in noisy environments and in correctly localizing a sound (Kidd et al., 2005; Culling and Lavandier, 2021). Particularly in the case of informational masking, in which similar sound signals are mixed, e.g., target speech with background speech, spatial release from masking by localizing the different sound sources is important for following and understanding the target signal (Papesh et al., 2017). Two important cues enable this localization: the interaural level difference (ILD), naturally created by the acoustic shadow of the head (see Figure 1A), and the interaural time difference (ITD) created by the differences in arrival time of sound signals at the left and right ear. The superior olive complex (SOC), a brainstem nucleus and the first site of binaural integration, is the key structure in binaural signal integration for the computation of ILDs. Neurons in the lateral part of the SOC, the LSO, receive excitatory input from the ipsilateral cochlear nucleus and inhibitory input from the contralateral cochlear nucleus via a transmitter, the medial nucleus of the trapezoid body (MNTB), see Figure 1B. This interaction of ipsilateral excitatory and contralateral inhibitory inputs creates a dynamic balance of ILD values encoding the location of a sound source (Tollin, 2003). Thereby, it is important that the sensitivity of the sensors and neuronal excitability are similar for both sides and across frequencies to enable a reasonable encoding of ILD (Darrow et al., 2006).

As a second key factor in sound-in-noise perception (e.g., the cocktail party effect), several adaptation mechanisms are implemented along the auditory processing pathway. Such adaptation mechanisms start at the sensory level, by changing the sensitivity of hair cells and neuronal spike-rate adaptation to constant sounds, and reach all the way to the cortex (Willmore and King, 2023; Khalighinejad et al., 2019; Angeloni et al., 2023). Adaptation is thereby driven by local information, like input and signal statistics, or by efferent feedback from downstream areas based on processing performance and goals (Dahmen et al., 2010; Angeloni et al., 2023; Guinan, 2018; Lopez-Poveda, 2018). A major area for producing such efferent feedback signals to the sensory stage, i.e., the hair cells and auditory nerve fibers in the cochlea (see green connections in Figure 1B)depicts the SOC (Zhao et al., 2022; Guinan Jr, 2006; Frank and Goodrich, 2018). It has been shown that this feedback can change the gain and dynamic range of the sensors and neuronal responses, enhance signal contrast, and improve signal perception in background noise (Guinan, 2018; Lopez-Poveda, 2018). Furthermore, regarding sound source localization, efferent feedback is discussed to improve interaural sensitivity for ILD encoding (Darrow et al., 2006), as schematically shown in Figure 1C. Furthermore, some brainstem areas exhibit adaptation to sound-source locations (Stange et al., 2013; Gleiss et al., 2019; Lingner et al., 2018), enabling better spatial hearing. For example, adaptation in dependence of the mean or variance of the ILD signal distribution is observed, combined with a shifting of the mean of the ILD response or the perceptual sensitivity (Dahmen et al., 2010) (see Figure 1D). The latter refers to balancing the left and right inputs to the brain stem area to improve sensitivity.

Efferent feedback connections can be ipsilateral as well as contralateral, as indicated by the green lines in Figure 1B, and the feedback signals can be excitatory, inhibitory, or modulatory (Guinan, 2018; Lopez-Poveda, 2018). While the topology and signal types are well resolved, the underlying mechanisms of efferent feedback and how it can improve hearing, e.g., for speech-in-noise or sound source localization, remain under debate. Furthermore, only a few models exist, describing the mechanisms of efferent feedback (Farhadi et al., 2023). Since efferent feedback directly tunes the sensory input to the following stages, it is particularly unclear how it affects different processing tasks and stages. For example, if efferent feedback is excited by noise to improve sound-in-noise perception, does this change in sensory properties affect the performance of the sound source localization stage at the same time?

While biological systems have evolved sophisticated mechanisms for robust, efficient, and real-time sound perception, particularly in dynamic and noisy environments, artificial auditory systems still struggle to match their performance (Patman and Chodroff, 2024). Integrating bio-inspired algorithms and using hardware-based, neuromorphic implementations can improve sound-in-noise recognition and increase the system's efficiency (Araujo et al., 2020; Liu et al., 2014). In this context, artificial cochleae were developed to replicate biological hearing processes. Such bio-inspired devices utilize nonlinear filtering and frequency decomposition techniques to improve the perception of speech and environmental sounds as well as efficiently encode important sound features as amplitude or frequency (van Schaik and Liu, 2005; Jiménez-Fernandez et al., 2017; Wang et al., 2015; Liu et al., 2014; Yang et al., 2016; Hamilton et al., 2008; Thakur et al., 2014; Xu et al., 2018b; Singh et al., 2019; Nouri et al., 2015). Furthermore, neuromorphic implementations of sound source localization algorithms have been developed (Oess et al., 2020b; Schoepe et al., 2023; Schmid et al., 2023) that analyze and encode ILDs or ITDs efficiently and in real time. Combining artificial cochlea and ILD/ITD analysis with phonotaxis, i.e., turning a robotic head or body movement towards a sound source, can be efficiently implemented (Schoepe et al., 2023; Reeve et al., 2005), which in turn can help improve sound perception in noisy environments.

In some of these systems, adaptation was introduced to adjust gain, dynamic range, or sensitivity, see, e.g., (Kiselev and Liu, 2021), which can improve localization (Oess et al., 2020a). However, existing systems often exhibit limited adaptability, rendering them susceptible to variations in signal-to-noise ratio and dynamic acoustic conditions. In particular, the sensing element, i.e., the microphone, is typically not adaptable, and thus its sensitivity cannot be tuned. Recently, we introduced an adaptive microelectromechanical system (MEMS) cochlea (Lenk et al., 2023) that comprises artificial hair cells obtained via MEMS-based transducers in combination with electronic feedback. Thus, this artificial cochlea can mimic the frequency selectivity, adaptability, and dynamic range of its biological counterpart. Tuning the feedback parameters can enhance the response to low-signal-to-noise conditions, increase the dynamic range, and highlight/extract important sound features such as sound onset (Durstewitz et al., 2022; Ved et al., 2024). All the above-mentioned implemented adaptations are based on signal statistics and do not include information from downstream processes, unlike efferent feedback. Nevertheless, implementing a sound coding strategy based on the medial olivocochlear reflex, which is part of the efferent system, in cochlear implants can improve speech intelligibility in the presence of speech maskers (Lopez-Poveda et al., 2017).

Our goal is to integrate efferent feedback into a neuromorphic sound source localization system to study the effect of sensor adaptation driven by different types and topologies of efferent feedback on the bio-inspired detection of ILD. By incorporating such an adaptive element, we aim to replicate the adaptivity and efficiency of biological auditory processing, particularly for sound source localization, and to improve the long-term performance of neuromorphic hearing devices. To achieve this, our bio-inspired architecture couples an artificial cochlea with adaptive feedback from lateral superior olive (LSO) neurons, enabling dynamic, real-time optimization of sound source localization (see Figure 1). In this approach, the MEMS cochlea (Lenk et al., 2023) is combined with a computational LSO model (Oess et al., 2020a). The model, composed of conductance-based neurons, integrates excitatory input from the ipsilateral ear with inhibitory input from the contralateral ear to encode ILD cues. Crucially, the neuron model can be deployed directly on neuromorphic hardware (Schmid et al., 2023), making the combination with the neuromorphic cochlea an architecture inherently suited for energy-efficient, embedded auditory sensing. The newly implemented adaptive feedback loop forms a bidirectional mechanism where LSO neuronal activity influences the tuning of the MEMS cochlea, which in turn influences LSO activity. By closing the loop between cochlear sensing and LSO-driven adaptation, the system mimics key aspects of the biological auditory pathway, enabling dynamic adjustments in response to changing acoustic environments. In the following study, we first explore whether efferent feedback from LSO neurons to the artificial cochlea can be used to tune the cochlea's sensitivity and the LSO neuron's response, thus enabling a wider perceptual range of input. Second, we study whether the efferent feedback in this system can help overcome sensor differences, e.g., due to fabrication tolerances, thereby balancing the interaural sensitivities. Finally, we demonstrate that gain adaptation can lead to temporal encoding of inputs in LSO neurons. This has two beneficial consequences: first, the system is highly responsive to onsets of sounds, thus enabling fast reactions to stimuli, and second, the time course of the response can be used as additional encoding of the ILD, if the sensitivity of the LSO neuron is not high enough. This allows the system to maintain a wide range of input sensitivity, enabling quick reaction to a stimulus while also allowing high precision in stimulus direction when necessary.

In the following section, we present the system architecture and the different types and topologies of efferent feedback. Then, the ILD analysis by the system is described. In sec. 3, the results from measurements and simulations of the system with and without efferent feedback will be presented and discussed. Finally, we will present our conclusions from this study.

## 2 Methods

### 2.1 System setup

#### 2.1.1 Architectural overview

**Figure 2:**
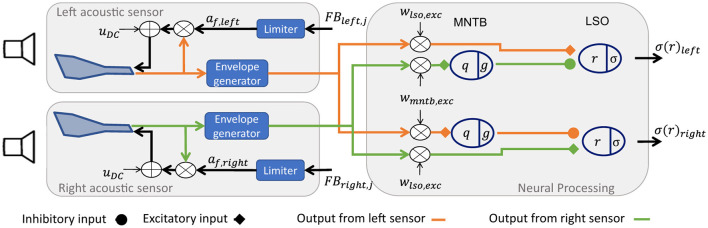
System architecture overview. Simplified architectural overview of the simulation system with two speakers, two acoustic sensors (left and right), and one neural processing stage. Each acoustic sensor consists of a MEMS sensor with pre-amplification, an envelope generator connected to the neural processing stage, and feedback to the sensor based on amplification factor *a*_*f*_(*t*) and bias offset *u*_*dc*_, as well as the input from neural processing *FB*_*i*_ and a feedback limiter. The neural processing stage consists of MNTB and LSO neurons with membrane potential (*q, r*) and activation function (*g*, σ). Their input is scaled and is only excitatory for the MNTB neuron, and excitatory and inhibitory for the LSO neuron. σ is the final LSO output of the system.

Inspired by the sound source localization of humans using ILDs, the here implemented system (Figure 2) detects ILD from sound sources by a two-stage processing: (i) speaker input is fed to two bio-inspired acoustic sensors, modeling the left and right ear input and cochlear processing. (ii) Then, a subsequent neural processing stage combines the sensing outputs from both sides and forms an ILD estimate. Both parts, the bio-inspired sensors and the neural processing stage, are explained in detail in the following Subsections 2.1.2 and 2.1.3, respectively. Finally, the output of the neuronal processing is used to drive the feedback to the sensor stage to model efferent feedback. The different feedback algorithms are detailed in Section 2.1.4. How the system is investigated for ILD analysis within different experimental settings is described in Section 2.2.

#### 2.1.2 Acoustic sensor

Each acoustic sensor consists of a MEMS cantilever with pre-amplification electronics, an envelope generator, and feedback to the sensor. The MEMS sensor is a silicon cantilever with an integrated piezo-resistive readout to transduce the beam bending due to sound excitation into a voltage signal *u*_*s*_(*t*). Due to its resonant operation, each MEMS cantilever is mostly responsive to its resonance frequency and thus acts as a band-pass filter with linear transfer characteristics. Its resonance frequency depends on the geometric dimensions of the cantilever. Furthermore, an aluminum heater is integrated into the MEMS as a thermomechanical actuator. This allows actuating the cantilever and introducing additional beam bending, which enables tuning of the sensor (Rangelow et al., 2017; Lenk et al., 2023). Using a simple feedback mechanism


$$\begin{array}{l} u_{act}\left(t\right)=a_{f}\left(t\right)\cdot u_{ac}\left(t\right)+u_{dc} \end{array}$$ (1)

consisting of the amplified, high-pass filtered sensor response *u*_*ac*_(*t*) and a bias voltage *u*_*dc*_, tuning of the sensitivity, bandwidth, and linearity of the cantilever's response is possible (Ved et al., 2024). Particularly, if *a*_*f*_(*t*) is tuned close to a critical point *a*_*crit*_, the cantilever behaves like a small-signal amplifier with compressive transfer characteristics. At *a*_*crit*_ the system undergoes a Hopf bifurcation leading to autonomous oscillations (Johann Rolf and Meurer, 2023). *a*_*crit*_ can be analytically calculated using the system parameters like filter time constants and transfer factor (Lenk et al., 2023; Johann Rolf and Meurer, 2023)). This tunability can be used to extract sound features, such as on- and offset (Durstewitz et al., 2022, 2024) and, in conjunction with the frequency decomposition, for signal pre-processing. The latter helps to improve speech processing in noisy conditions (Johny et al., 2024).

The model description of the cantilever's response is based on the Euler-Bernoulli beam theory and was derived in Roeser et al. (2016); Lenk et al. (2023). In this mass-normalized model equation (terms are expressed in units of acceleration $\frac{\text{m}}{{\text{s}}^{2}}$), the deflection *x*(*t*) of the free end of the cantilever due to the thermal-mechanical actuation α_*s*_θ(*t*) and an external force ${\tilde{F}}_{ext}\left(t\right)$ generated by the sound level can be described by the following second-order ordinary differential equation (ODE):


$$\ddot{x}(t)+\frac{\omega_{0}}{Q_{0}}\dot{x}(t)+\omega_{0}^{2}x(t)=\alpha_{s}\theta (t)+{\tilde{F}}_{ext}(t)$$ (2)

where ω_0_ = 2π·*f* with the resonance frequency *f*, *Q*_0_ denotes the quality factor, α_*s*_ is the transfer factor from temperature to deflection, and θ(*t*) is the temperature difference between the beam and its ambient temperature.

The change in the temperature difference is caused by the actuation and can be written as


$$\begin{array}{l} \dot{\theta }\left(t\right)+\beta_{s}\theta \left(t\right)=\gamma_{s}{\left(\frac{\tanh u_{act}\left(t\right)}{R}\right)}^{2} \end{array}$$ (3)

with the time constant β_*s*_, the transfer factor from voltage to temperature γ_*s*_, and the applied actuation voltage *u*_*act*_(*t*), limited by tanh, to the thermo-mechanical heater with the heater resistance *R*. The electrical output of the cantilever *u*_*s*_(*t*) = κ_*s*_·*x*(*t*), determined by the deflection *x*(*t*) and a factor κ_*s*_ due the piezo-resistive elements and pre-amplification, is high-pass filtered


$$\begin{array}{l} {\dot{u}}_{ac}\left(t\right)+\frac{u_{ac}\left(t\right)}{\tau_{hpf}}={\dot{u}}_{s}\left(t\right) \end{array}$$ (4)

with the time constant τ_*hpf*_ to remove the static beam deflection. Next, the upper envelope *u*_*env*_ is extracted by rectifying and low-pass filtering


$$\begin{array}{l} \tau_{lpf}{\dot{u}}_{env}\left(t\right)+u_{env}\left(t\right)=|u_{ac}\left(t\right)| \end{array}$$ (5)

with time constant τ_*lpf*_. This *u*_*env*_(*t*) is the output of the sensing stage and the input to the neural processing stage. The high- and low-pass filter equations are derived by applying Kirchhoff's laws to simple resistor-capacitor circuits and can be used for hardware-based implementations of the filter functions. The filtered beam response *u*_*ac*_(*t*) is further used for feedback to the sensor by generating the actuation signal *u*_*act*_(*t*) as given by Equation 1. Here, depending on the experimental setting (described in Section 2.2) *a*_*f*_(*t*) is either a constant value or given by the input *FB*_*i*_ from the neural processing stage to model the efferent feedback. This is described in detail in Section 2.1.4.

#### 2.1.3 Neural processing

The architecture of the neural processing stage comprises LSO and MNTB neurons and is based on the model in Oess et al. (2020a). This model captures the main temporal dynamics of how a circuit of representative biological LSO and MNTB neurons computes their membrane potential and firing rate activity and is based on experimental evidence. To clarify how the MNTB and LSO neurons contribute to extracting ILDs from the envelope of the sensor output *u*_*env*_, the underlying mathematical formulations are presented below. The model utilizes single-compartment conductance-based equations to describe the evolution of the neurons' membrane potentials.

The state of the MNTB neuron is described by its membrane potential *q* and evolves according to


$$\begin{array}{l} \tau_{q}\dot{q}\left(t\right)=-\alpha_{q}q\left(t\right)+\beta_{q}s_{q}\left(t\right) \end{array}$$ (6)

with the time constant τ_*q*_, the leakage part α_*q*_*q* with the decay rate α_*q*_ and the excitatory input obtained from the scaled sensor response *s*_*q*_(*t*) = *u*_*env*_(*t*)·*w*_*mntb, exc*_ and an additional scaling factor β_*q*_. The firing rate of the MNTB neuron is computed from the membrane potential by


$$\begin{array}{l} g\left(q\right)={\left[q\left(t\right)\right]}_{+}=\max\left(q\left(t\right),0\right), \end{array}$$ (7)

i.e., using a half-wave rectification as an activation function. The LSO neuron membrane potential *r* evolves according to a conductance-based model description.


$$\begin{array}{l} \tau_{r}\dot{r}\left(t\right)=-\alpha_{r}r\left(t\right)+\left(\beta_{r}-r\left(t\right)\right)s_{r}\left(t\right)-\left(\gamma_{r}+\kappa_{r}r\left(t\right)\right)g\left(q\left(t\right)\right). \end{array}$$ (8)

Here, the rate of change is the sum of three terms: a decay term −α_*r*_*r*, an excitatory term (β_*r*_−*r*(*t*))*s*_*r*_(*t*) and an inhibitory term −(γ_*r*_+κ_*r*_*r*(*t*))*g*(*q*(*t*)). The decay term with the decay rate α_*r*_ leads to an exponential decay toward a resting potential of 0 in the case where no excitatory or inhibitory input is present. The excitatory input *s*_*r*_(*t*) = *u*_*env*_(*t*)·*w*_*lso, exc*_ comes from the sensor envelope and is constrained by the force term (β_*r*_−*r*(*t*)) with an upper saturation level described by the reversal potential β_*r*_. The inhibitory input *g*(*q*(*t*)) is the output of the MNTB neuron and is constrained by the force term −γ_*r*_−κ_*r*_*r*(*t*). This term bounds the membrane potential by a lower saturation level described by a reversal potential of $-\frac{\gamma_{r}}{\kappa_{r}}$ with the parameters γ_*r*_ for subtractive- and κ_*r*_ for divisive-type of neural inhibition. Finally, the firing rate of the LSO neuron is obtained from the sigmoidal activation function σ(*r*(*t*))


$$\begin{array}{l} \sigma \left(r\left(t\right)\right)=\frac{1}{1+\exp\left(-\left(r\left(t\right)-\beta_{\sigma }\right)\cdot \alpha_{\sigma }\right)} \end{array}$$ (9)

with the parameters α_σ_ and β_σ_ for shaping the steepness and offset of the sigmoid, respectively.

#### 2.1.4 Efferent feedback configurations

**Figure 3:**
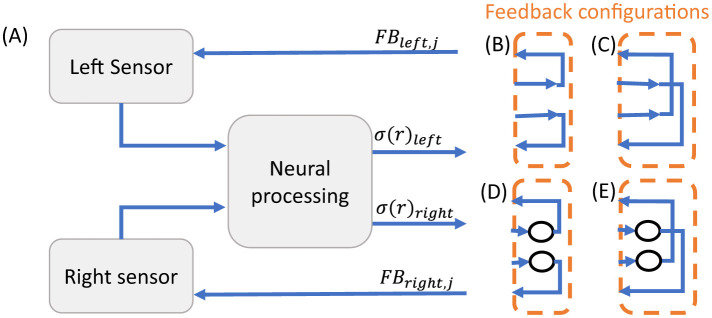
Implemented feedback configurations from the LSO-MNTB subsystem onto the acoustic sensors. **(A)** Simplified system interface representation with feedback to the left and right acoustic sensor (*FB*_*left, j*_, *FB*_*right, j*_), outputs of the sensors to the neural processing stage and outputs from the neural processing stage (σ(*r*)_*left*_, σ(*r*)_*right*_). **(B–E)** Implemented feedback configurations from LSO neuron to acoustic sensor: a positive (non-inverted)feedback to the sensor on the ipsilateral side in (B) and on the contralateral side in **(C)**, negative (inverted) feedback to the sensor on the ipsilateral side in **(D)** and on the contralateral side in **(E)**.

As previously mentioned, efferent feedback modulates the gain, dynamic range, and shifts the mean of the ILD response. Inspired by this mechanism used for adaptation, the output of the neuronal processing stage is used to change the feedback to the sensor, as indicated in Figure 2. Different configurations are conceivable. Here, we focus on four configurations that (i) cover a broad range of possible system interactions (positive versus negative feedback loops and same-sided versus opposite-sided projections), and (ii) can be easily and efficiently realized in hardware. The four configurations (Figures 3B–E) are determined by in- and outputs of the interface shown in Figure 3A. Based on the sign of the feedback, two configurations from a positive feedback loop (B,C), while the other two describe a negative feedback (D,E). The projection side defines another discrimination criterion. Here, ipsilateral refers to feedback from the LSO neuron to the sensor on the same side, i.e., left-to-left side or vice versa. Contralateral is the feedback from the LSO neuron to the sensor on the opposite side.

These feedback configurations are implemented according to


$$\begin{array}{l} a_{f,i}\left(t\right)=k_{inv}\cdot min\left(FB_{i,j}\left(t\right),k_{max}\right)\cdot a_{f,crit} \end{array}$$ (10)

with the feedback strength *FB*_*i, j*_∈{0, 1} given by the output σ(*r*) of the neuronal processing stage as


$$\begin{array}{l} FB_{i,j}\left(t\right)=\sigma_{j}\left(r\left(t\right)\right) \end{array}$$ (11)

Here, *i, j* denote the sides of the sensor and the LSO neuron, respectively, i.e., left or right. Feedback connections with *i* = *j* are ipsilateral projections, and *i* ≠ *j* are contralateral projections. To obtain positive or negative feedback, the inversion factor *k*_*inv*_ = ±1 was introduced. Thus, non-inverted feedback refers to positive feedback. The sensor feedback strength *a*_*f*_(*t*) is given in relation to the critical point *a*_*f, crit*_ as


$$\begin{array}{l} a_{f}\left(t\right)=k_{a}\left(t\right)\cdot a_{f,crit}, \end{array}$$ (12)

Since this relationship defines the linearity of the dynamics and the increase in gain. The larger the gain factor *k*_*a*_(*t*), the larger the gain of the sensor, and for *k*_*a*_(*t*)>0.9, nonlinear dynamics can be observed, as described in Section 2.1.2 and Lenk et al. (2023); Ved et al. (2024). Finally, using the *min* function, *a*_*f*_(*t*) is restricted to ≤ *k*_*max*_·*a*_*f, crit*_ to avoid unwanted oscillations due to the efferent feedback and additional nonlinear effects. A value of *k*_*max*_ = 0.95 was obtained empirically from the simulations.

### 2.2 Experimental setups for ILD analysis

After describing the three parts of the implemented system in the previous sections, we will now introduce the experimental settings used to study the system's performance for ILD analysis. These experiments are intended to obtain a general understanding of the range of possible system behaviors. Thereby, we focus on two relevant sources of influence:

- **Influence of sensor responses**. ILD analysis by the neural processing stage is directly influenced by the acoustic sensors' response properties. We particularly investigate the influence arising from different, constant feedback strengths *a*_*f*_(*t*) = *a*_*f, i*_ = *const* and differences in the sensor properties on the left and right sides due to, e.g., fabrication tolerances.
- **Influence of efferent feedback configurations**. Different efferent feedback mechanisms between the neural processing stage and the acoustic sensors are conceivable. We investigate the influence of such feedback configurations on the steady-state and maximal responses of LSO neurons and on the temporal evolution of the LSO response.

The experimental setting for studying the sensor influence is described in Section 2.2.1 and the corresponding results are given in Section 3.1. The experiments for the investigation of different efferent feedback configurations are presented in Section 2.2.2 and their corresponding results in Section 3.2. How these results are obtained from the outputs of the LSO neurons and the sensors is detailed in Section 2.2.3. The experiments in all cases are based on simulations of the models descried above for the neuronal processing stage and utilize either measured or modeled sensor responses to sound. All models and analyses have been implemented in Matlab^TM^ 2024a, and differential equations are solved with ode15s, a multistep solver based on the numerical differentiation formulas (NDFs). The simulation parameters are presented in Table 1.

**Table 1:** Parameters for simulations described in Sections 2.2.1 and 2.2.2.

**Parameter**	**Value**
**Acoustic sensor**	
Resonance frequency, *f*_0_	1·10^3^ Hz
Quality factor, *Q*_0_	50
Transfer factor, α_*s*_	19.2 m s^-2^K^-1^
Time constant, β_*s*_	1.0066·10^3^ s^-1^
Transfer factor, γ_*s*_	16.2·10^6^ KΩ^2^s^-1^V^-2^
Heater resistance, *R*	15 Ω
Piezo and Amplification factor, *k*_*p*_	0.602·10^6^ Vm^-1^
Time constant of high-pass filter, τ_*hpf*_	1·10^−3^ s
Feedback offset, *u*_*dc*_	−100·10^−3^ V
Critical feedback factor, *a*_*f, crit*_	0.03236
Time constant of low-pass filter, τ_*lpf*_	5·10^−3^ s
**Sound signal**	
**Neural processing stage**	
**MNTB parameters**	
Time constant, τ_*q*_	25·10^−6^ s
Leak constant, α_*q*_	2
Multiplication factor, β_*q*_	1 V
Multiplication factor, *w*_*mntb, exc*_	2 (Section 2.2.1), [200, 400, 750] (Section 2.2.2)
**LSO parameters**	
Time constant, τ_*r*_	25·10^−6^ s
Leak constant, α_*r*_	1
Excitatory reversal potential, β_*r*_	1 V
Multiplication factor, *w*_*lso, exc*_	1 (Section 2.2.1), 100 (Section 2.2.2)
Inhibitory reversal potential, γ_*r*_	3 V
Divisive inhibition constant, κ_*r*_	4
Sigmoid parameter, α_σ_	20 V^-1^
Sigmoid parameter, β_σ_	0.2 V
Sound signal slope, α_*l*_	0.5
Sound signal offset, β_*l*_	0.5
Sound signal frequency, *f*	1·10^3^ Hz

#### 2.2.1 Setup for ILD analysis without efferent feedback to study sensor influence on LSO neuron response

The first set of experiments investigates the impact of the sensor properties, particularly the feedback strength *a*_*f*_ and the deviation between two sensors, on subsequent processing in the system. To this end, we record the pre-amplified and high-pass filtered responses *u*_*ac*_(*t*) of two different acoustic MEMS sensors to sounds generated by a Genelec 8010AP-6 loudspeaker. For measurement, a data acquisition unit with 100 kS/s sample rate was used, and sound signals were generated in Matlab and applied to the loudspeaker via a Babyface Pro FS. Sound signals were recorded simultaneously with a reference microphone beyerdynamic MM 1, to obtain the sound amplitudes of the microphone recordings.

Two sets of measurements were performed: first, the resonance frequency of each sensor was determined; second, the sound response for different feedback strengths and sound amplitudes was acquired. To determine the resonance frequency, the sensors were placed at a distance of 1 m from the loudspeaker, and a pure-tone frequency sweep was played. The resulting sensor response, pre-amplified by a factor of 1,000 and high-pass filtered with a cut-off frequency of 159 Hz, was converted into a frequency response by FFT, and the highest-amplitude peak was extracted as the resonance frequency. For the here used MEMS cantilevers, the extracted resonance frequencies are 950 Hz and 986 Hz. In the second measurement, each sensor was excited separately with a pure tone sound at its resonance frequency with different sound amplitudes. Besides the sound amplitude, also the feedback strength *a*_*f*_ was varied, i.e. *k*_*a*_ = [0, 0.2, 0.4, 0.6, 0.8], while *u*_*dc*_ was kept constant at −100 mV. Then, the maximal amplitude of the sensor signal was calculated and plotted against the RMS sound amplitude to obtain the transfer characteristics.

Finally, to study the influence of feedback strength and the differences in sensor properties on the ILD analysis, the sensor responses were used as input for simulations of the neuronal processing stage. In detail, Equations 1–4,10,11 were neglected in the model, and the measured data were directly input as *u*_*ac*_(*t*) for Equation 5. To check the influence of the different sensor characteristics and feedback, two cases were simulated: (i) The sensor response dataset is the same for the left- and right sensor in the simulations, using the measurements of sensor 1 with the resonance frequency of 950 Hz and (ii) the dataset used for the left sensor comes from sensor one (resonance frequency of 950 Hz) and the dataset used for the right sensor comes from sensor two (resonance frequency of 986 Hz). The first condition investigates how a real sensor would affect subsequent system behavior across different feedback levels, while the second condition investigates how the introduction of sensor discrepancies, e.g., from manufacturing tolerances, would further affect system behavior. The results are described in Section 3.1.

#### 2.2.2 Setup for ILD analysis including efferent feedback from LSO neurons

In the previous section, acoustic MEMS sensors were measured and used as input to the simulation of envelope extraction and subsequent processing by the LSO-MNTB subsystem. To investigate an adaptive feedback for the sound source localization the outputs of the neural processing stage (σ(*r*)_*left, i*_, σ(*r*)_*right, i*_) need to feed back into either the ipsi- and contralateral acoustic sensors (via *FB*_*left, i*_, *FB*_*right, i*_; see Figure 3A). To study this, the complete system was simulated to test a broader range of configurations and observe their effects on system behavior. A synthetic sound signal was generated, fed into the sensor model as *F*_*ext*_, and the sensor response was used as input to the MNTB and the LSO neurons, with an additional input to the LSO neuron based on the MNTB neuron output. The output of the LSO neuron is then used to change the feedback to the sensor, i.e., *FB*. To study the ILD dependence, different ILD levels *lvl* are applied, and the LSO response σ to the different levels is the final measure. Thereby, the sound for the left and right sensor is synthetically generated for a fixed ILD level *ILD*, a fixed slope α_*l*_, and an offset β_*l*_ according to


$$\begin{array}{l} F_{ext,j}=\left(\pm \alpha_{ILD}\cdot ILD+\beta_{ILD}\right)\cdot \sin\left(\omega t\right),j\in \left\{left,\;right\right\} \end{array}$$ (13)

This equation satisfies the constraint of a constant sum of left and right sound levels for different ILD levels, i.e., *F*_*ext, right*_+*F*_*ext, left*_ = α_*ILD*_*ILD*+β_*ILD*_−α_*ILD*_*ILD*+β_*ILD*_ = *const*. This approach filters out cues related to the overall loudness and distance. Since in these simulations we used identical left and right sensors, the sound frequency is the same on both sides. Furthermore, there were no differences between the sides included in these studies, i.e., the simulation parameters were identical for both acoustic sensors, for both MNTB neurons, and for both LSO neurons. An overview of the parameters is presented in Table 1. These simulations were conducted for the four configurations of efferent feedback to study their influence on ILD extraction and the sensitivity of LSO neurons. Additionally, the potential impact of the excitatory-inhibitory balance between LSO and MNTB neurons on ILD extraction was studied. To this end, simulations with different values for the MNTB neuron scaling factor *w*_*mntb, exc*_ and the LSO neuron scaling factor *w*_*mntb, exc*_ have been conducted for a broad set of input ILD ranges. The results are presented in Section 3.2.

#### 2.2.3 Analysis

The architecture and model equations are explained in detail in the previous sections, together with the parameters in Table 1 used to execute the simulations. To analyze the influence of sensor properties, feedback configuration, and excitatory-inhibitory balance, the output of the LSO neurons was studied in terms of steady-state behavior, temporal dynamics, and sensitivity to ILD for a simulation time of 1 s. The initial condition of the system state was determined by performing an initialization step. Therefore, the system was simulated for 2 s in an open-loop setting to determine the initial equilibrium point. Afterwards, the system's feedback configuration has been established, and an input stimulus has been provided.

The steady state response σ_*eq*_ was calculated as the average value of the last 0.333 s for simulations in Section 3.1 and 0.1 s for all the other simulations in Section 3.2 for both LSO neurons and feedback configurations, separately. To get the impact of both steady state LSO outputs over the whole ILD range, the difference Δσ_*eq*_ = σ_*right*_−σ_*left*_ is calculated.

To study the temporal behavior of the system, the LSO response over time for a fixed set of parameters is analyzed in section 3.2.2. Therefore, the time *TT*_90_ of the first occurrence of 90% of the LSO response maximum σ(*r*(*t*_*max*_)) is computed to inspect characteristics of the transient behavior. Additionally, the temporal evolution of the system is studied by extracting responses σ(*r*(*t*)) for non-overlapping temporal slices *t*∈[*t*_*n*_, *t*_*n*_+Δ*t*] of Δ*t* = 0.1 s. The average activity per temporal slice is computed subsequently. Response curves for specific time slices across the temporal evolution are then obtained by computing average values for each ILD level and combining them into a curve.

The sensitivity of the LSO response is computed by numerical derivation of the steady-state response σ_*eq*_(*ILD*) with respect to ILDs δσ/δ*ILD*, using a central finite differences scheme.

## 3 Results

### 3.1 Influence of sensor properties on neuronal processing of ILDs (without efferent feedback)

**Figure 4:**
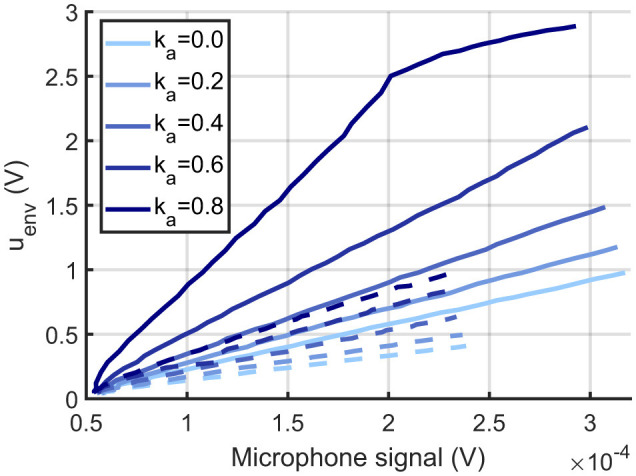
Measured MEMS sensor responses. Extracted envelope response of two MEMS sensors vs. microphone signal amplitudes for pure tone sound signals with each sensor excited at its resonance frequency (left sensor 950 Hz solid line and right sensor 986 Hz dashed line) and for different feedback strengths *a*_*f*_/*a*_*crit*_ = *k*_*a*_ = 0, 0.2, 0.4, 0.6, 0.8.

The first set of experiments investigated the feasibility of performing the ILD analysis using measurements from fabricated MEMS sensors together with the neural processing stage model. The response of the two sensors (resonance frequencies 950 Hz and 986 Hz), i.e., their transfer characteristics, is shown in Figure 4. A largely linear relationship is observed between the reference microphone amplitude, as a measure of sound amplitude, and the envelope signal *u*_*env*_(*t*) of the sensors. Thereby, increasing the feedback strength increases the sensor gain, as described earlier, and only linear characteristics are observed due to the restriction *k*_*a*_ ≤ 0.8. Furthermore, because of differences in sensor properties, the two sensors exhibit different response amplitudes for similar sound amplitudes. The highly linear relationship between the microphone signal and the stationary input provides a characteristic that can be easily leveraged by subsequent processing stages. Additionally, the monotonic relationship with constant feedback indicates that, in principle, there exists a clear control dimension for tuning the sensor's response curve. On the one hand, these circumstances indicate that the MEMS sensors can be used as meaningful transducers for the input sound signals. On the other hand, there are clear influences of differences in sensor properties (geometry, fabrication tolerances, etc.) on the sensor response. The effect size of these tolerances is of the same order as the sensor's operating range. Thus, one can compensate only for a certain amount of difference using feedback, limiting the number of sensors that can be used.

**Figure 5:**
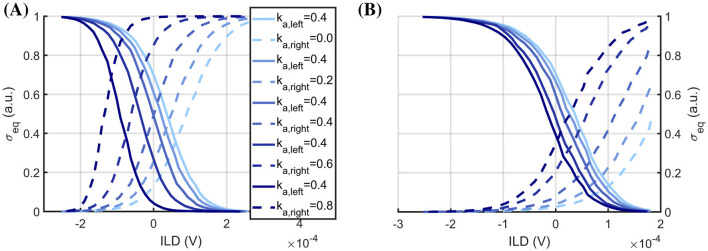
System ILD response characteristics. LSO response profiles across input ILDs for excitation-inhibition balance 1:2 for different feedback settings *k*_*a*_ = 0, 0.2, 0.4, 0.6, 0.8 and same acoustic MEMS sensor data for left (solid line) and right (dashed line) in **(A)** and for data from two different sensors in **(B)**. The point of equal activation (σ_*eq, left*_ = σ_*eq, right*_) shifts depending on the feedback setting and the pairing of identical or different sensors (see Section 3.1 for details).

In a second step, the obtained envelope signals were used as input into the neural processing stage for ILD analysis. Here, either the measurements from one MEMS sensor were used to model both the left and right sensors in the model, or the measurements from both MEMS sensors were applied to stimulate the neural processing stage (Figures 5A, B, respectively). The system was run in an open-loop setting, i.e., without direct feedback from the neural processing stage to the acoustic sensing stage. Instead, constant feedback to the acoustic sensing stage was applied with different constant feedback factors *a*_*f, i*_(*t*) = *a*_*f, i*_ = *const*. Thereby, the feedback strength for the left sensor was kept constant while that for the right sensor was varied to study its effect.

From Figure 5, it can be observed that the response curves of the left and right LSO neurons resemble the typical sigmoidal tuning curve expected from their biological counterpart (cf. Figure 1). This observation holds independent of the use of measurements from only one MEMS sensor (Figure 5A) or two MEMS sensors with fabrication-based differences (Figure 5B) and independent of the utilized constant feedback factor *a*_*f, i*_. However, only in the case of input from one MEMS sensor, the LSO responses are symmetric for the left and right neurons. Furthermore, the influence of the feedback constants is common in both simulations with either one or two MEMS sensors. The response curves shift depending on the left-right balance of the constant feedback factors *a*_*f*_. In particular, the point of equal amplitude between the left and right LSO, i.e., the intersection point between both curves, is shifted further to more negative ILD values and larger LSO activities σ for larger *a*_*f*_ constants on the right acoustic sensing stage. Similarly, the intersection points are moved further toward negative ILD values and smaller σ values for larger *a*_*f*_ constants on the left acoustic sensing stage. In this way, the left-right balance of feedback strengths can be used to tune the sensitivity more toward one side or the other.

Notably, for the simulated case of identical MEMS sensors and equal constant feedback (one MEMS sensor measurement used as left and right input; Figure 5A, *a*_*f, left*_ = *a*_*f, right*_ = 0.4), the point of equal amplitude perfectly matches an ILD value of zero. Thus, for a balanced setup with equal feedback strength on the left and right sides, the system would produce a balanced output. Instead, for the case of two differing MEMS sensors and equal constant feedback (one MEMS sensor measurement per input side; Figure 5B, *a*_*f, left*_ = *a*_*f, right*_ = 0.4) the point of equal amplitude is off center (here, shifted towards the left, *ILD* < 0). Therefore, the system would produce an imbalanced output due to the sensor differences, e.g., due to fabrication. Importantly, though, a balanced system output can be re-established by counterbalancing via different feedback strengths (stronger constant feedback to the right than to the left; Figure 5B, *a*_*f, left*_ = 0.4, *a*_*f, right*_ = 0.8)). This highlights how specifying, or learning, specific feedback strengths per sensor could be used to calibrate the overall system in light of fabrication-based differences of the MEMS sensor to balance the input to the neuronal stage. Given the results, this approach would be possible, since the order of magnitude at which feedback strength *a*_*f*_ can alter the response profile is the same as the effect size of the sensor differences.

However, this local feedback *a*_*f*_ is assumed to adapt to signal statistics, such as reducing the feedback for large sound amplitudes for protection reasons or reducing the response to constant sounds to highlight important features or reduce redundant information (sensory adaptation). In such cases, one possibility to still balance the input to the neuronal processing stage is efferent feedback from the neuronal processing stage, thereby dynamically adjusting the feedback strengths *a*_*f*_ to tune the sensor response profiles to match each other. Another benefit of dynamically tuning the sensor's response profiles via efferent feedback arises when sensor response profiles degrade, e.g., due to aging or temperature effects. In this case, dynamic adaptation could keep the response profiles matched, achieving system robustness. Possible system behaviors resulting from efferent feedback for the task of ILD analysis are discussed in the following section.

### 3.2 Influence of efferent feedback on system response

The second set of experiments investigates the possible system behavior if an efferent feedback is introduced from the neuronal processing stage to the sensor stage (see Figures 2, 3). The focus here is on the range of system responses, its temporal response, stability, and ILD sensitivity and tuning.

#### 3.2.1 Configuration-dependent LSO neuron response characteristics

**Figure 6:**
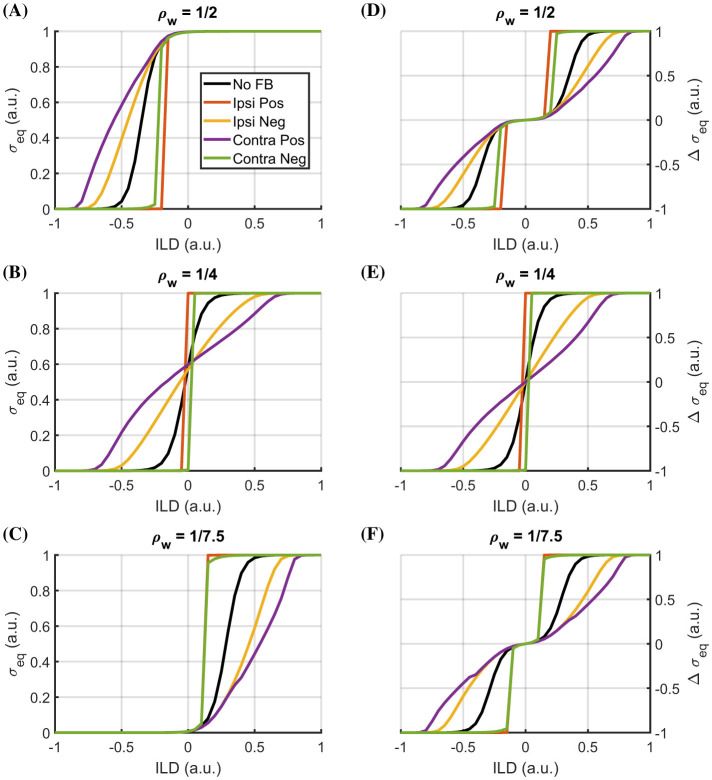
System ILD response characteristics at equilibrium. Response profiles of right LSO neuron across input ILDs for different excitation-inhibition balance levels ρ_*w*_ [left column, **(A–C)**] and respective right-left difference profiles [right column, **(D–F)**]. Five different feedback configurations are depicted: without feedback from the LSO neuron (No FB, black; constant *a*_*f*_), positive ipsilateral (Ipsi Pos, red; *k*_*inv*_ = +1, *i* = *j*), negative ipsilateral (Ipsi Neg, orange; *k*_*inv*_ = −1, *i* = *j*), positive contralateral (Contra Pos, purple; *k*_*inv*_ = +1, *i* ≠ *j*), and negative contralateral (Contra Neg, green; *k*_*inv*_ = −1, *i* ≠ *j*). In all cases, the steady-state response σ_*eq*_ is shown.

To study the influence of the efferent feedback onto the system characteristics, the LSO response at equilibrium was extracted across the range of tested ILD values (*ILD* = [−1, 1]), as shown in Figures 6A–C using different excitation-inhibition balances ρ_*w*_ = *w*_*LSO*_/*w*_*MNTB*_ = 1:2 (A), 1:4 (B), and 1:7.5 (C) (cf. Section 2.2.2 for details). Here, only the response of the right LSO neuron is shown, since both curves (left and right LSO neuron responses) are symmetric. Subsequent processing for sound source localization based on the ILD could use either the response from one side or a combination of the responses from both sides. Thus, to give an idea of the input for the latter case, the difference between left and right response σ_*right*_−σ_*left*_ is shown in the right column of Figures 6D–F). In all graphs, the black curve represents the case without efferent feedback, whereas the colored lines represent the four different feedback configurations: positive ipsilateral (red), positive contralateral (purple), negative ipsilateral (orange), and negative contralateral (green line).

For all curves, a monotonic increase in the LSO neurons' response amplitude with increasing ILD can be observed (Figure 6, right column). Changing the ratio between inhibitory input (*w*_*MNTB*_) and excitatory input (*w*_*LSO*_) for the LSO neuron shifts its sensitive range. Here, with an increasingly stronger inhibitory influence, the location of the slope shifts from the contralateral towards the ipsilateral side (from Figures 6A–C). If no efferent feedback is applied, the overall shape of the response curve (σ vs. ILD) is thereby preserved. Beyond these common properties of monotonic increase and the shift in sensitivity due to the excitation-inhibition balance, the different feedback configurations have distinct effects, mainly altering the shape of the response curves and their relative positions compared to the case without feedback. Two configurations increase sensitivity while decreasing the ILD range within which the LSO neuron is sensitive, compared to the case without efferent feedback. This can be attributed to either a direct effect increasing the excitatory input to the LSO neuron by the amplifying feedback to the sensor on the ipsilateral side (ipsi positive feedback) or an indirect effect via damping the sensor response on the contralateral side due to the negative feedback and thus decreasing the inhibitory influence (contra negative feedback). Conversely, the two other configurations lead to a shallower slope with a broader sensitive range - either directly via the damping (negative) feedback to the ipsilateral side, reducing the excitatory influence (ipsi negative feedback), or indirectly via the positive (amplifying) feedback to the contralateral side, increasing the inhibitory input to the LSO neuron (contra positive feedback).

Furthermore, an interaction exists between the excitation-inhibition balance and the effect of feedback configuration. The configurations with the lower sensitivities (ipsi negative and contra positive) are affected the most, i.e., they exhibit the strongest shift of their response curves to the contralateral side for ρ_*w*_ = 1/2 and the ipsilateral side for ρ_*w*_ = 1/7.5. The opposite pattern is visible for the feedback configurations, yielding an increase in sensitivity. Their sensitive response regions move less strongly away from the center when the excitation-inhibition ratios change. In this context, the lateralization, i.e., the strength of this shift, is most pronounced for the feedback configuration with positive feedback to the contralateral side (purple line). These results demonstrate that it is possible to tune both the sensitivity and the lateralization of LSO neurons using efferent feedback at the sensing stage.

Computing the difference between the left and right LSO output largely compensates for lateral displacements of the sensitive regions independent of the feedback configuration and excitation-inhibition balance, but preserves the different effects on the slope of the response curve (Figures 6D–F). Due to these slope changes, the feedback configuration can result in either more analog ILD coding (purple and orange curves) or a more digital response with two or three saturation regimes for configurations with increased sensitivity (red and green curves). The latter could be used to estimate the direction of the sound source, enabling a fast reaction, such as turning the head towards it. Conversely, analog coding could support sound-stream segregation if localization and sound analysis are combined to enable, for example, listening to a specific sound source while remaining aware of the overall environment.

In sum, the impact of feedback configuration and excitation-inhibition balances on the system's processing can be leveraged for technical applications. With these design dimensions, it is possible to largely shift and tune the system's processing to better reflect the concrete circumstances of a current context. Additionally, despite the combination of two nonlinear dynamical systems, a stable response is obtained. Nevertheless, the temporal behavior exhibits interesting properties, which are discussed in the next section.

#### 3.2.2 Temporal response behavior

**Figure 7:**
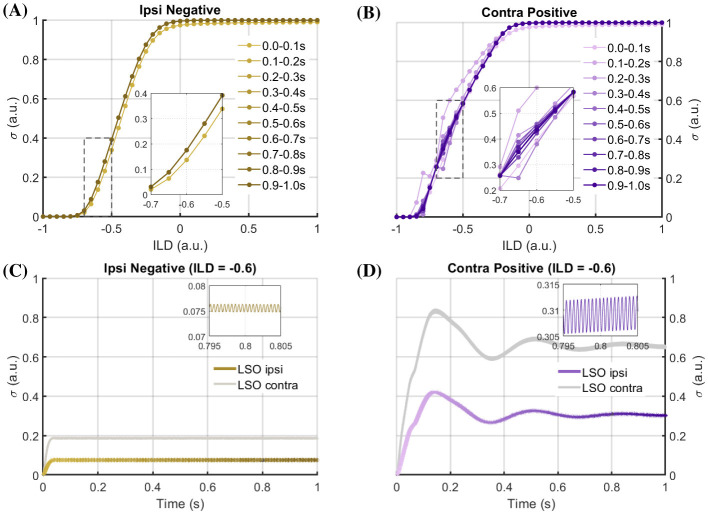
LSO temporal response characteristics. LSO response profiles across input ILDs for different temporal periods (color coded) after stimulus onsets for the case of negative ipsilateral feedback **(A)** and positive contralateral feedback **(B)** for an excitation-inhibition balance factor of ρ_*w*_ = 1/2. C, D: exemplary temporal LSO response profiles for the respective cases **(C, D)**. Gray response profiles depict the contralateral LSO response for the respective case (not shown in **(A)** and **(B)**). Insets depict details from the main plots. Colors for the type of feedback configuration match the ones from Figure 6.

The above results revealed how the system's behavior depends on the feedback configuration and the excitation-inhibition balance for constant input sound levels, and how to analyze the stable (converged equilibrium) responses. Beyond these response characteristics, the system exhibits rich temporal dynamics. Depending on the specific ILD, the feedback configuration and excitation-inhibition balance, a transient response phase is visible in the LSO response profile (cf. Figures 7C, D for exemplary behaviors). For example, in Figure 7C, the negative feedback onto the ipsilateral sensor yields a damping effect and a fast saturation of the LSO response to the equilibrium value, after a short transient phase. For this case, the overall LSO neuron response σ(*ILD*) does not change over time, as demonstrated by the responses obtained after different time intervals in Figure 7A. A different pattern can be observed for a configuration with positive feedback to the contralateral side, which increases the sensor response and thus the inhibitory input to the LSO neuron Figures 7B, D). In this case, the LSO neuron response oscillates around the equilibrium value before settling at it. The transient time to reach the equilibrium value is much longer than in the previously discussed case. These effects are differently pronounced for different ILD values (Figure 7B). Overall, these examples provide hints on how the system's temporal response characteristics are influenced by the discussed design choices. Viewing the system as an oscillator, it either becomes more damped Figures 7A, C) or less damped (more oscillatory) Figures 7B, D) depending on the feedback configuration and parametrization. Such a tendency towards more or less volatility could be exploited in technical setups, making the system more robust to fluctuations or more sensitive to them, depending on application demands. Furthermore, the oscillatory response enables both fast and slow responses to sound localization, as described above. The first maximum of σ provides a fast but coarse estimate of the sound source, which could be used for rapid danger detection and rapid movement towards or away from the sound source. Furthermore, it would initially create a stronger differentiation between localizations of different sound sources. The equilibrium value, on the other hand, provides a slower but more accurate estimate of the ILD, which might be more important for understanding and segregating sounds in noisy environments.

**Figure 8:**
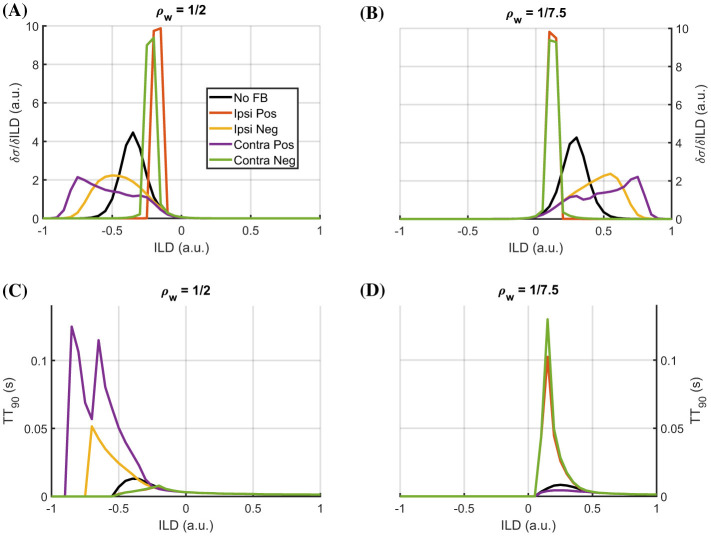
Sensitivity and timing of LSO response characteristics for different ILDs. Extracted gradient of ILD system responses for different feedback configurations and an excitation-inhibition balance level of ρ_*w*_ = 1/2 in **(A)** and ρ_*w*_ = 1/7.5 in **(B)**. In **(C)** and **(D)**, the time to reach 90% of maximum LSO amplitude is shown for different ILDs and the same excitation-inhibition balance levels as in **(A)** and **(B)**.

Additionally, a close relationship exists between the time *TT*_90_ it takes the system to reach its transient maximum (i.e., 90*%σ*_*max*_), its sensitivity, i.e., the slope, of the system for a given ILD value, and its lateralization (Figure 8). ILD ranges, in which the system is operated in a non-saturating regime (slope different from zero; Figure 8), exhibit a longer *TT*_90_ than the saturated ones. Interestingly, though, *TT*_90_ profiles deviate from the baseline configuration without feedback (no FB) only if the σ_*eq*_ of the respective feedback configuration is larger than that of the case without feedback. This typically occurs on the more contralateral side of the LSO response curve in the no-feedback condition. For which feedback configurations and which ILDs this holds, depends strongly on the excitation-inhibition balance ρ_*w*_. The feedback configurations with a smaller σ_*eq*_ than the no- feedback case show only minor deviations from the respective baseline profiles and lead to overall shorter *TT*_90_ values.

Such a behavior could serve different purposes—either as a *confidence indicator*, or as an *additional coding dimension*. In terms of a *confidence indicator*, longer detection times (interval until transient maximum is reached) could signal subsequent processing units, that the sensitive range of the LSO neuron shifts towards the more contralateral side. Thus, the detection might be less confident, and potentially more weight should be given to the opposite LSO neuron. In this case, the *TT*_90_ could be used as a fast indicator during the transient phase of the response to determine whether the input's ILD range likely falls into the range for which the specific LSO neuron is sensitive or not. This indication could be used to adaptively re-tune the LSO neuron with the factors identified above (i.e., feedback configuration and excitation-inhibition balance) to adjust the sensitivity range to the currently relevant ILD range. In terms of a *additional coding dimension*, the response time *TT*_90_ itself can be used as another encoding of the ILD alongside the amplitude response code for the more contralateral sounds. Particularly for the cases with rather small sensitivities *dσ*/*dILD*, using the *TT*_90_ value could improve the resolution in ILD detection.

## 4 Discussion

This study presents a novel approach to neuromorphic auditory processing by integrating adaptive bio-inspired acoustic sensors, as a model of the cochlea, with an efferent feedback mechanism, driven by the neural processing stage. The latter is inspired by superior olive processing for sound localization based on ILD, realized by LSO and MNTB neurons. The bio-inspired acoustic sensor models the processing and sensing of the cochlea, particularly frequency decomposition and (nonlinear) amplification of the input. The neural feedback dynamically adjusts the sensing properties, such as gain, bandwidth, and linearity, by changing the strength of the local feedback to the sensor. This local feedback models the impact of outer hair cells in the cochlea and the sensitivity tuning of the acoustic neuron fibers at the synapse with the inner hair cell. As demonstrated by measurements from two different sensors, adjusting this local feedback to the sensors changes ILD sensitivity only slightly but can help overcome differences in the sensing stage between the left and right sides, which might arise from fabrication tolerances, aging, and environmental influences. However, since efferent feedback can also be driven by sources outside the LSO, this would influence LSO-based ILD detection by shifting the balance between left and right inputs. Efficiently processing multiple efferent feedback sources remains an open problem, necessitating the development of appropriate mechanisms to overcome current limitations.

The MEMS cochlea and the utilization of its feedback component, together with the dynamical multi-stage cross-hemispherical processing, are key components of the proposed system architecture and set it apart from many previous studies. While other impressive approaches to neuromorphic sound source localization exist, many utilize artificial preprocessing of sound source data (Glackin, 2010; Wang et al., 2018; Zhang et al., 2024; Ware et al., 2025), ultrasonic transducers (Moro et al., 2022), silicone cochlea chips (Chan et al., 2010), and FPGA cochleas (Xu et al., 2018a; Schoepe et al., 2023) instead of the MEMS cochlea with tunable sensors. Notably, these other approaches typically use inter-aural timing difference (ITD) as the coding paradigm to determine correlates of sound source location, and subsequently pair it with one or multiple stages of feedforward processing. The proposed mechanism here chooses inter-aural level difference (ILD) as the main coding paradigm. Furthermore, it offers interesting avenues for time-domain encoding that depend on the sensitivity range of the specific feedback configuration. Specifically, these feedback projections from the neural processing stage back to the cochlear sensor, with their possibility for different configuration types, are a distinctive element of the proposed model and are not found in any of the other approaches mentioned above-an element from which additional power and complexity for the computation of sound information can be drawn.

Inspired by the variety of connections in the human efferent systems, we studied four different feedback connections as combinations of whether the feedback projects to the ipsi- or the contralateral cochlea sensor and whether it affects the sensor's sensitivity positively or negatively. These feedback configurations correspond to a certain extent to established efferent pathways of the Superior Olivary Complex. Namely, ipsilateral and contralateral feedback reflect the influence of medial olivocochlear fibers, which modulate outer hair cells and dynamically adjust cochlear gain on the same or opposite side, thus enhancing interaural contrast (Lopez-Poveda, 2018; Guinan, 2018). Thereby, an inhibiting effect is observed for sounds in quiet environments, similar to negative feedback in our model. In noisy environments, an enhancement of cochlear response for target sounds can be observed (Lopez-Poveda, 2018), which corresponds to positive feedback in our sensor system in the model. Similarly, lateral olivocochlear efferent connections influence type-I auditory nerve fibers ipsilaterally and contralaterally, whereby enhancing and reducing afferent sensitivity rather than mechanical gain. However, the lateral olivocochlear efferents are less clearly resolved due to the difficulty in stimulating the unmyelinated fibers (Lopez-Poveda, 2018). Since the feedback to the MEMS cochlea sensor modifies the overall sensor gain, i.e., the overall artificial cochlear response, both effects (outer hair cell modulation and nerve fiber sensitivity change) can be modeled by modulating the feedback gain. Thus, all four modeled configurations map onto plausible Superior Olivary Complex connections, since the biological efferent system contains both ipsi- and contralateral projections, and both gain-enhancing and gain-reducing effects. This explicit alignment strengthens the relevance of our feedback configurations as simplified but biologically grounded abstractions of cochlear efferent control. The influence of the different efferent feedback connections was studied for different excitation-inhibition balances, i.e., input gains for MNTB and LSO neurons' excitatory input. All configurations yield stable responses, and the monotonic increase in response amplitude with increasing ILD toward the respective LSO neuron's ipsilateral side aligns well with the general working principle of the biological system. This shows that implementing efferent feedback into a technical system with MEMS-based acoustic sensing units and biologically inspired ILD processing can be feasible and successful in general.

The choice of feedback configuration and excitation-inhibition balance poses itself as a critical design dimension of the system. Changing the feedback configuration tunes the sensitivity of the LSO response, and changing the excitation-inhibition balance shifts the sensitive ILD range. The sensitivity of the steady state responses for the right LSO neuron can be sorted from low to high based on the feedback configuration: (i) positive contralateral, (ii) negative ipsilateral, (iii) no Feedback, and (iv) negative contralateral, which is similar to the positive ipsilateral case. In the latter two cases, due to the more complex interplay between the sensor and neural processing stages, the response curve differs from the sigmoidal shape typically observed in biological systems, and in the absence of efferent feedback. However, this change in shape can be used to encode ILDs differently. In the case of strongly increased sensitivity, a binary response of the LSO neuron is obtained, whereas a reduction in sensitivity in the other two feedback configurations allows for a more analog encoding of ILDs. Depending on the application, one can thus switch between analog and binary encoding of the ILDs.

Regarding the shift in sensitive ILD range, an increase in the inhibition from the contralateral acoustic sensor, i.e., decreasing the excitation-inhibition balance, leads to a shift of the sensitive ILD range to more positive ILDs for the right LSO neuron. Thus, the difference between the two LSO neuron responses remains centered around *ILD* = 0 because different excitatory or inhibitory parameters influence the left and right LSO neuron responses equally, owing to the symmetric architecture. Besides the case of ρ_*w*_ = 1/4, a range with almost no sensitivity to ILD changes occurs around *ILD* = 0. In fact, the difference in LSO neuron response appears quite similar for ρ_*w*_ = 1/2 and ρ_*w*_ = 1/7.5, although the single LSO neuron response shifts strongly. Thus, depending on the processing of the subsequent stages, reading out one LSO neuron might be sufficient (case ρ_*w*_ = 1/4) or, if the difference of LSO neurons is used, it is possible to obtain a stable response despite shifting the sensitive range of neurons. In the latter case, the sound source localization from the difference of LSO neuron responses refers to a relative position, whereas the single LSO neuron response could provide an absolute position. The difference computation between the left and right LSO outputs serves as a simple linear example for further processing of the system output. It outlines the possibility of recombining different elements of the representation to retain some aspects of the coding principle, e.g., differences in sensitivity (slope) between feedback configurations, while discarding others, e.g., the lateral shift of the response curves. In the simple example presented here, this study performed particularly well because the left and right acoustic sensing and neural processing stages exhibited symmetric response behavior under identical parametrization. Furthermore, the choice of feedback configuration switches the combined response between a more analog and a more digital representation, which would be advantageous for different applications. In this sense, tuning to specific ILD ranges and sensitivities enables dynamic, adaptive sound source localization.

While all feedback configurations lead to stable LSO neuron responses, the temporal responses yield further information about the ILDs and feedback configurations. Here, feedback configurations that resulted in stronger responses than in the case without feedback further yielded oscillatory responses about the steady-state value. This was particularly pronounced for ILDs on the more contralateral side. The first peak of these responses (before the steady-state) is reached much later than the steady-state response in the other cases. This provides an additional temporal coding of ILD and signals to subsequent stages, if feedback were applied to shift the sensitive range toward more contralateral ILDs.

While stable responses were observed in this study, systems without limitations on feedback strength, fast varying input signals, or different parameter settings might yield unstable configurations. Thus, examining the system from a control-theoretic perspective could strengthen the analysis of the observed effects. In particular, linear stability analysis around fixed points, bifurcation analysis, and small-signal gain analysis, or frequency-response characterization, could be applied (Khalil, 2014), which is useful for uncovering computational properties of recurrent neural networks in general (Sussillo and Barak, 2013). Additionally, as a more recent technique, contraction analysis can be used to investigate exponential incremental convergence of state-space trajectories to one another without explicitly considering a fixed point (Lohmiller and Slotine, 1998); see (Tsukamoto et al., 2021) for a recent tutorial. In individual sub-systems, related investigations were already fruitful. For example, Brosch and Neumann (2014) studied the mathematical and stability properties of an excitatory-inhibitory (E-I) neuron pair to show how it behaves under different input and parameter combinations. This E-I pair is structurally conserved by the LSO-MNTB circuit and thus could serve as a meaningful basis for sparking further stability analyses in the larger Cantilever-LSO-MNTB system. Further foundational understanding of the system's closed-loop characteristics will help with broader application of the idea. For example, in the neuromorphic setting, this analysis could prove useful for understanding how small differences (e.g., from quantization artifacts during conversion to the chip, or from drift on analog chips) affect the system's functioning.

In principle, the joint system of biologically inspired acoustic sensors and neurons of the auditory pathway can be implemented in neuromorphic hardware. Combining sensor measurements with the neuron models provided a first proof-of-principle. The neuronal stage, i.e., LSO and MNTB neurons, had previously been implemented on neuromorphic hardware, specifically TrueNorth and Spinnaker (Schmid et al., 2023). In the neuromorphic implementation, the adaptation of the feedback can be used to overcome differences in the sensors due to, e.g., aging or fabrication tolerances. Adding efferent feedback enables tuning sensitive ranges and sensitivity and, thus, the implementation of adaptive sound source localization. The dynamic adaptation of the sound source localization can be used in a closed-loop scheme to minimize localization error. Expected advantages include a fast, noise-robust sound source localization system that can easily handle dynamic and moving sound sources. If combined with a motor control, closed-loop systems for navigation based on sounds could be implemented for real-time and efficient operation, e.g., in robot systems. Furthermore, modulatory control of neuronal responses, e.g., due to GABA receptor adaptation, could be added to enhance the adaptability and tuning of sound source localization and incorporate adaptation due to previous stimuli (Oess et al., 2020a). Additionally, learning capability for the excitation-inhibition balance as well as the feedback parameters could be introduced to improve the left-right balance of the system and address challenges due to other sources of efferent feedback, which yield a different tuning of sensors as required for sound source localization.

The results were obtained for two acoustic sensors, which limits the localization task to sounds near their resonance frequencies. To expand the implementation for complex sounds, the system can be easily scaled up using multiple acoustic sensors with different resonance frequencies, together with multiple neuronal processing stages. Owing to the band-pass filter characteristics of the sensors, noise sources with different spectral components are effectively filtered, leading to a noise-robust system. The effect of the feedback varies under different environmental noise conditions. Positive feedback to the sensor will decrease its bandwidth and increase the filtering properties. Negative feedback decreases the sensor response when the overall noise level is high, preventing saturation of the sensor and subsequent stages. Thus, the feedback strength could be dynamically adjusted to dampen or strengthen the sensor response for each side depending on the respective noise level. Asymmetric feedback could further increase this effect. Noises influencing the same spectral components can be reduced by decomposing the sound into streams and selectively adjusting the sensitivity to a specific stream by adjusting the feedback settings to dampen or amplify the sensor responses.

The findings highlight the potential of bio-inspired feedback control for next-generation auditory sensors, with promising implications for hearing augmentation and machine listening applications. Potential applications of this technology include advanced hearing aids, robotic auditory systems, and enhanced speech recognition devices. By leveraging the principles of biological adaptation, our approach opens new possibilities for developing intelligent auditory processing systems that can operate effectively in real-world scenarios.

## Data Availability

The custom-developed MATLAB simulation code is available in the repository https://github.com/Victory6921/cantilever-sensors-w-neuronal-processing-stage and the data are publicly available at the following link: https://doi.org/10.5281/zenodo.18244293.
